# Psychometric Properties of the Diener Satisfaction With Life Scale With Five Response Options Applied to the Colombian Population

**DOI:** 10.3389/fpubh.2021.767534

**Published:** 2022-01-13

**Authors:** Begoña Espejo, Marta Martín-Carbonell, Irene Checa, Yadid Paternina, Martha Fernández-Daza, Juan D. Higuita, Angela Albarracín, Ara Cerquera

**Affiliations:** ^1^Department of Behavioral Sciences Methodology, School of Psychology, Universidad de Valencia, Valencia, Spain; ^2^Psychology Program, Universidad Cooperativa de Colombia, Santa Marta, Colombia; ^3^Psychology Program, Universidad Cooperativa de Colombia, Medellín, Colombia; ^4^Psychology Program, Universidad Pontificia Bolivariana de Bucaramanga, Bucaramanga, Colombia

**Keywords:** satisfaction with life scale, response options, psychometric properties, confirmatory factor analysis, well-being assessment, measurement invariance, structural equation modeling

## Abstract

**Introduction:** The Satisfaction with Life Scale (SWLS), developed by Diener, Emmons, Larsen, and Griffin in 1985, comprises five items with seven response options in terms of agreement–disagreement. Recently, there has been a suggestion to reduce the response options of the SWLS to optimize its applicability in different cultural contexts.

**Objective:** The study aims to assess the psychometric properties of the SWLS with five response options in the Colombian population. Specifically, we studied the dimensionality, invariance by gender and age (among a group of adolescents and emerging adults under 25 years and a group of adults of intermediate age and established adulthood under 59 years), convergent validity (with optimism), and divergent (with pessimism) and concurrent validity with other measures of well-being (flourishing, positive, and negative affects).

**Methodology:** This project was a cross-sectional study using a non-probabilistic sample of the general population. Participants were included if they identified themselves as Colombian and were at least 18 years of age. The final sample comprised 1,255 participants. The average age was 25.62 years (SD = 8.60) ranging from 18 to 67 years of age, and 35.8% of the participants were men. In addition to SWLS, we used the Flourishing Scale (FS), Life Orientation Test-Revised (LOT-R), and Scale of Positive and Negative Experience (SPANE).

**Results:** Cronbach's alpha coefficient (0.842), composite reliability (0.851), and average variance extracted (0.537) showed very good values. CFA was conducted to test the one-dimensional structure of FS, showing excellent goodness of fit [$\chi_{\left(5\right)}^{2}$ = 15.774, *p* < 0.001, CFI = 0.992, TLI = 0.985, RMSEA = 0.042, 90% RMSEA CI (0.020, 0.066), and SRMR = 0.016]. The correlations calculated among life satisfaction (SWLS) with flourishing (FS), positive and negative affects (SPANE), optimism, and pessimism (LOT-R) were statistically significant and as expected. Configural, metric, and scalar invariance across gender and age were confirmed. Percentiles were provided for the total score and for age.

**Conclusions:** The SWLS with five response options has adequate psychometric properties in the Colombian population, and the use of this version (with 5 response options) is recommended due to its greater applicability.

## Introduction

In recent decades, politicians and governments have shown an increased interest in evaluating well-being (1). Among other reasons, this is due to the accumulated evidence about its impact on health (2), in academic performance (3) and in labor (4), as well as its value to inform government decisions and evaluate programs aimed at promoting mental health and quality of life in risk groups (5). Research shows that the concept of subjective well-being is multidimensional (6). Among the constitutive components of well-being, satisfaction with life has been identified as a distinct construct that involves a cognitive and global assessment of quality of life as a whole. It has also been conceptualized as the self-assessment of an individual's quality of life according to the comparison between their current state and their standard of what is desirable (7).

According to various authors (5, 8), the most widely used measure for this research is the Satisfaction with Life Scale (SWLS), originally developed by Diener et al. (9). These authors suggest that the scale allows access to the positive side of the individual experience and that it emphasizes self-assessment itself, because the person can establish the basis of their evaluation by choosing the domains that they will take into account when assessing their life, regardless of their emotional state (10). The SWLS is based on the theory of global satisfaction originally proposed by Sumner (11), who conceptualized global satisfaction as a positive attitude toward life itself. This implies that it is an evaluation of all areas and stages of life, which includes both the affective and cognitive aspects, according to the person's expectations.

The SWLS consists of five items with seven response options in terms of agreement–disagreement on a Likert scale ranging from 1 to 7. While the authors of the scale (10) did not provide normative data, they proposed cut-off points that correspond directly to the seven response categories: 31–35, very satisfied; 26–30, satisfied; 21–25, slightly satisfied; 20, neutral; 15–19, slightly dissatisfied; 10–14, dissatisfied; and 5–9, very dissatisfied.

There is sufficient evidence of the validity of the satisfaction with life construct and the SWLS scale, verified, first by their ability to detect differences associated with objective conditions and different life circumstances (12); second, by their correlations with measures not based on self-reporting (13); third, by their association with genetic and physiological variables (14); fourth, by significant changes in scores associated with major life events; and finally by the predictive value of suicidal behaviors (15, 16). SWLS scores have also been shown to positively correlate with health variables and negatively with emotional symptoms, negative thoughts, and coping strategies such as experiential avoidance (17).

Since it was introduced, the SWLS has been used in hundreds of studies (13, 15). It has been validated in numerous languages, such as French (18), German (19), Portuguese (20), Turkish (21), Chinese (22), and even sign language (23). Its psychometric properties have been explored with a wide variety of populations, including adolescents, the elderly, and patients with different health problems (24).

However, modifications to the SWLS response options have recently been recommended in order to optimize its applicability in different cultural contexts (25). This goes in line with previous studies that have shown that offering too many response options could be problematic for people with a low cultural level (26) or generate confusion and boredom in respondents who may find it difficult to distinguish subtle differences between categories (27).

Since the use of Likert scales is intended to adequately represent a construct or continuous latent variable, the question of the optimal number of response alternatives and the effect of categorizing continuous variables becomes particularly relevant. Some authors showed in a study that no psychometric advantages were apparent as of six-response options (28), but this is a subject still under discussion (29). On the other hand, and as a result of a systematic review of published literature on this issue (30), it was concluded that it is best to use five response options. In general, according to studies of the International Test Commission, when data are one-dimensional the best fit is achieved when working with four to six categories (31). In addition, the easier it is for the user to respond to this type of measure, the greater its applicability is, allowing its use to be extended to people with limited comprehension or communication skills, who are often excluded from studies, such as the visually or hearing impaired, people with a low education or those who are illiterate, and people with cognitive problems (26). On the other hand, an invariance study was carried out specifically for the SWLS with Italian and African populations (25). It found that the scale may not be sensitive when it comes to detecting low levels of satisfaction with life, so the authors recommended using fewer response options, especially for the South African population.

There are several adaptations of the SWLS in Spanish: one can be found in public domain on the website of Ed Diener (https://eddiener.com/), the main author of the original scale. It was translated by José A. Reyes-Torres. However, we also found several reports using back translation (32–36). These adaptations differ both in the wording of the items and in their order of presentation, as well as in the number of options and the text of the responses.

Several studies in Ibero-American populations have used the SWLS with five instead of the seven options from the original scale, e.g., in Spain (32, 33, 35, 37), in Chile (38), in Peru (39), in Costa Rica (40) and in Puerto Rico (41). In Mexico it was conducted a validation study of the scale with three-response options (disagree, intermediate, and agree) in a national sample of 13,220 adults above the age of 50 years, finding adequate internal consistency, criterion validity, and confirmation of the one-factor structure (42). In Colombia, different variants of the SWLS have been used in different populations and contexts (43–45). This motivated another authors (46) to conduct an initial validation study with a sample of 121 University students using a version with seven response options (32), but drafted in terms of satisfaction-dissatisfaction (from very dissatisfied to very satisfied), which limits its applicability in cross-cultural studies.

A recent research (47) have recently studied the psychometric properties of Atienza's version (36) in a Colombian sample but with seven response options, instead of the five whose translation was validated by Atienza et al. They have not clarified the origin of the translation used for these seven-answer options. This goes against the recommendations of the International Test Commission for adapting instruments that have been developed in other contexts (31, 48). For the version they studied, Ruiz et al. reported adequate internal consistency and corroborated that the unifactorial model had a very good fit, significant correlations, went in the expected direction with other measures of well-being, and showed metric and scalar gender invariance and with a Spanish sample. However, they did not report normative data that could facilitate its application in contexts where individual differences need to be established.

For these reasons, the present study aimed to evaluate the psychometric properties of Diener's SWLS in the Spanish version by Atienza et al. (35, 36) with Colombian population and using five response options. In addition to being easier to answer for our population, it has been and is widely used in Ibero-American contexts. We investigated whether the one-dimensionality of the instrument with five response options was maintained, as well as the gender invariance. We also studied the age invariance of the SWLS in Colombians, which had not been done previously and constitutes a topic of interest given then reports on the absence of age invariance in other samples (24). We were interested in corroborating the concurrent validity of the SWLS with measures of optimism and pessimism because of existing evidence regarding their relationship (49–51) and their convergent validity with other measures related to well-being, such as prosperity and positive and negative affects (51–53). Finally, another objective was to obtain information to interpret the scores of the Colombian population.

## Methods

### Participants

The present project was a cross-sectional study using a non-probabilistic sample of the general population. Participation in the survey was completely anonymous and voluntary, and no participant received any type of financial compensation for it. Participants were included if they identified themselves as Colombian and were at least 18 years old. The final sample comprised of 1,255 participants. The average age was 25.62 years (*SD* = 8.60) ranging from 18 to 67 years, and 64.5% of the participants were female. People who had completed University or graduate studies formed the majority (42.9%) along with those who had completed high school (41.2%), 12.9% had completed secondary school, and only 2.7% of the sample had completed or partially attended primary education; 75.5% were single, 22% were married or had an intimate partner, and 2.5% were divorced or widowed. On the contrary, 43.9% were full-time students and 26.1% were in school and had sporadic or part-time jobs; regarding working status, 23.7% were employed or self-employed, 4.9% were unemployed, 1% were inactive, and 0.4% were retired.

### Measures

#### Satisfaction With Life Scale

This is an instrument designed to measure global cognitive judgment of satisfaction with one's life (9). For the present study, we used the Spanish adaptation with five response options by Atienza et al. (35, 36).

#### Flourishing Scale

This is an eight-item instrument describing important aspects of human functioning including positive relationships, feelings of competence, and having meaning and purpose in life (54). The instrument uses a seven-point Likert scale ranging from 1 (strongly disagree) to 7 (strongly agree). Total scores range from 8 to 56 with high scores indicating respondents viewing themselves in positive terms in important areas of functioning. This instrument was validated in a general sample of Spanish adults and showed an internal consistency of 0.85 (55). The present study uses the version validated in a Colombian sample, based on the previous Spanish version (56), with an internal consistency of 0.916 in the sample from this study.

#### Life Orientation Test-Revised

This questionnaire has been used to measure optimism and pessimism (57). The scale is comprised of ten items, four control items, three pessimism items, and three optimism items. Each item of the LOT-R is answered on a five-point Likert scale that ranges from 1 (strongly disagree) to 5 (strongly agree). The scores range from 0 to 12. Higher scores in both subscales indicate high optimism or high pessimism, respectively. This scale was validated in a sample of Colombian adults and showed good psychometric properties (58, 59). Internal consistency in this sample is 0.693 for the Optimism subscale, and 0.636 for the Pessimism subscale. Although some authors question Cronbach's alpha values lower than 0.70, this consideration should not be taken as a “golden rule,” especially due to the reduced number of items on the LOT subscales, since an alpha that is too high could lead one to think that, in reality, the three items measure the same indicator of the construct (60).

#### Scale of Positive and Negative Experience

This scale allows us to learn how the person evaluates the frequency with which they experience positive and negative feelings, as well as the balance of affections. To this effect, 12 adjectives organized in 2 subscales of 6 items each are used: 6 positive (SPANE-P) and 6 negative experiences (SPANE-N), measuring 3 general and 3 specific emotions in each subscale. The instrument uses a five-point Likert scale ranging from 1 (very rarely or never) to 5 (very often or always). Total scores range from 6 to 30, with high scores indicating a high positive or high negative affect. SPANE-P and SPANE-N can be subtracted to obtain a balanced measure (SPANE-B) that ranges from −24 to 24. In this study, the adapted version was used for a general sample of Colombians (61). The internal consistency in this sample was 0.811 for SPANE-P and 0.799 for SPANE-N.

### Procedures

Data from a larger study aimed at validating well-being scales in the Colombian population were used. Following the recommendations of Muñiz et al. (48), an initial qualitative pilot study was conducted. The pilot study's participants were selected using purposeful theoretical sampling serially until obtaining data saturation. In total, 14 people were included based on their willingness to collaborate and after ensuring they were Colombian adults (9 women and 5 men), with different education levels (8 people with a University education, 3 with high school diplomas, and 3 with primary school education), and between the ages of 18 and 81. The scale was responded to using paper and pencil and in an online version. The analysis of the participants' responses revealed that the wording of the items in the version for Spaniards was appropriate for the Colombian context and that the participants correctly understood the items in both versions (paper and pencil, and online). All participants stated that they understood the response options and had no difficulties in choosing the one they considered appropriate in both application formats.

Participants were recruited by different means (email, social networks, and also face-to-face). Data were collected online, with LimeSurvey, an open-source survey tool. When accessing the survey, an explanation of the study was presented, and participants had to read and accept an online informed consent before answering the survey. The study was approved by the Ethics Committee of the Cooperative University of Colombia, which guarantees that data collection complied with the Colombian Law of Data, ensuring confidentiality and anonymity.

### Data Analysis

Before studying the construct validity by means of a confirmatory factor analysis, the distribution of frequencies and percentages of the sociodemographic variables was investigated. The means and asymmetry coefficients of the items were checked, as well as the magnitude of the inter-item correlations (using Pearson's correlation coefficient). In addition, item-total corrected correlations were calculated for each item. A confirmatory factor analysis was performed to test the one-factor structure. The parameters were calculated using maximum likelihood robust estimation (MLR). While the nature of the data is ordinal, some studies suggest the use of MLR when the distribution of the data does not fit the normal curve and if there are five or more response options (62–64). In these situations, it can be assumed that the data is continuously distributed (65). The solution offered presents very little variability in the parameters (64), less biased standard errors, and good estimates of correlations between factors (66).

In order to study the fit of the data to the model, the Comparative Fit index (CFI), the Tucker-Lewis index (TLI), the root-mean-square error of approximation (RMSEA), and the standard-root-mean residual (SRMR) were used. Values of 0.90 for the CFI and the TLI, as well as values between 0.06 and 0.08 for the RMSEA and SRMR, indicate an acceptable model fit. Values above 0.95 for the CFI and the TLI and values below 0.05 for the RMSEA and SRMR indicate a good fit to the model (67–69). The factor measurement reliability of the SWLS was evaluated with Cronbach's alpha and with the Composite Reliability Index (CRI) (less biased than alpha) (70). The CRI is identical to the Omega coefficient (71) but more adequate when standardized factor loadings are used (72). In addition, the average variance extracted (AVE) (73) was calculated to evaluate the level of variance captured by the factor.

The measurement invariance by gender and age was evaluated by calculating three nested models that impose successive restrictions: configural, metric and scalar. Configural invariance test identical factor structures (i.e., the same number of factors and items and the same patterns of free and fixed loadings), metric invariance test equality of factor loadings, and scalar invariance test equality of factor loadings and thresholds. A configural model was first tested as a baseline model. In this model, all factor loadings and thresholds were estimated freely across groups. Unlike in models with continuous indicators, in models with categorical indicators with delta parameterization, metric invariance cannot be tested separately from scalar invariance (74, 75). Thus, a scalar invariance model was tested where equality constraints were simultaneously imposed on factor loadings and thresholds. Measurement invariance was examined by comparing the fit indices of the configural model and those of the scalar model. We used the cutoff criteria conventionally used. When sample size is adequate (total *N* > 300) and sample sizes are equal across the groups, a change of ≥-0.010 in CFI, supplemented by a change of ≥0.015 in RMSEA or a change of ≥0.030 in SRMR would indicate non-invariance (76). To study the measurement invariance according to age, the sample was divided into two groups: a group of adolescents and emerging adults (up to 25 years old) and a group of established adults (between 26 and 59 years old), in line with works by other authors (77). In Table 1 are shown the descriptive statistics for these two groups.

**Table 1:** Sociodemographic characteristics for the “emerging adults” group and for the “adults” group.

		**Emerging adults**		**Adults**	
		**Mean**	**Standard deviation**	**Mean**	**Standard deviation**
Age					
		21.07	2.12	34.86	9.37
		***N***	**%**	***N***	**%**
Gender					
	Male	267	59.47	182	40.53
	Female	574	71.22	232	28.78
Personal situation					
	Single	746	78.69	202	21.31
	Married or cohabiting	91	32.97	185	67.03
	Divorced	2	7.41	25	92.59
	Widowed	2	50.00	2	50.00
Educational level					
	Primary school studies	24	70.59	10	29.41
	Secondary school studies	137	84.57	25	15.43
	High school studies	428	82.79	89	17.21
	College studies				
	Undergraduate studies	250	56.18	195	43.28
Main activity					
	Studying	502	91.11	49	8.89
	Studying and working	237	72.48	90	27.52
	Working	64	21.48	234	78.52
	Unemployed, inactive or retired	38	48.10	41	51.10

*All differences are statistically significant (p <0.01), except for Widowed and Unemployed, inactive or retired*.

To study the convergent validity of satisfaction with life with other dimensions of well-being, Pearson correlations were calculated between the total scores of this scale and those of FS, SPANE (P and N), Optimism, and Pessimism. Pearson correlations from 0.20 to 0.39 were interpreted as weak; from 0.40 to 0.59 as moderate; and from 0.60 to 0.79 as strong. Above 0.80, the correlation was considered very strong. Finally, descriptive statistics and percentiles were provided for each age group and gender.

In order to carry out the confirmatory factor analysis and the invariance study, the statistical program Mplus 8.6 was used (74). So as to calculate the descriptive correlations among items and with criteria, Cronbach's alpha, the item-total corrected correlations, and the percentiles by groups, IBM SPSS 27 was used.

## Results

Table 2 shows the descriptive statistics of the items, the item-total corrected correlations, and the inter-item correlations. Note that the scores are not normally distributed, with values above the midpoint of the response scale predominating. In addition, the correlations among items present moderate values, except for the correlation between Items 3 and 4, which presents a high value.

**Table 2:** Descriptive statistics, item-total corrected correlations, and inter-item correlations among the items (Valid *N* = 1,222).

	**Item 1**	**Item 2**	**Item 3**	**Item 4**	**Item 5**
Mean	3.67	4.05	4.00	3.94	3.47
Mode	4	4	4	4	4
Standard deviation	1.025	0.899	1.016	0.987	1.266
Skewness	−0.747	−1.031	−0.945	−0.892	−0.412
SE of skewness	0.070	0.070	0.070	0.070	0.070
Kurtosis	0.035	1.140	0.353	0.429	−0.942
SE of kurtosis	0.140	0.140	0.140	0.140	0.140
Minimum	1	1	1	1	1
Maximum	5	5	5	5	5
Item-total corrected correlation	0.663	0.642	0.736	0.682	0.557
**Inter-item correlations**					
	**Item 1**	**Item 2**	**Item 3**	**Item 4**	
Item 2	0.539				
Item 3	0.590	0.571			
Item 4	0.587	0.514	0.658		
Item 5	0.435	0.458	0.513	0.437	

*SE, standard error; Item 1 = In most ways my life is close to my ideal; Item 2 = The conditions of my life are excellent; Item 3 = I am satisfied with my life; Item 4 = So far, I have got the important things I want in life; Item 5 = If I could live my life over, I would change almost nothing*.

Excellent fit values were found in the CFA for the one-dimensional model [$\chi_{\left(5\right)}^{2}$ = 15.774, *p* < 0.001, CFI = 0.992, TLI = 0.985, RMSEA = 0.042, 90% RMSEA CI (0.020, 0.066), SRMR = 0.016]. The factor loadings were all statistically significant (*p* < 0.001), ranging between 0.605 and 0.828. Cronbach's alpha was 0.842, the CRI was 0.851, and the AVE was 0.537. All of these values can be considered adequate.

Table 3 shows the results for the measurement invariance models by gender and age. The results show that the Colombian SWLS had scalar invariance by gender, and the fit of the one-dimensional model for men and women was good. As can be seen, ΔCFI, ΔRMSEA, and ΔSRMR values are lower than 0.010, 0.015, and 0.030, respectively. Thus, the latent mean values were fixed to zero for men and compared. No differences were found by gender (*b* = −0.008, *z* = −0.178, *p* = 0.859). Regarding the measurement of invariance by age, the results showed scalar invariance for the SWLS, and the fit of the one-dimensional model for emerging adults (until 25 years old) and adults (more than 25 years old) was excellent. Thus, the latent mean values were fixed to zero for emerging adults, showing that adults present more satisfaction (*b* = 0.155, *z* = 3.114, *p* = 0.002).

**Table 3:** Measurement invariance models of the SWLS by gender (reference group: men) and by age (reference group: under 25).

**Models for gender**	**χ^2^**	**df**	**Δχ^2^**	**Δgl**	**CFI**	**RMSEA**	**SRMR**	**ΔCFI**	**ΔRMSEA**	**ΔSRMR**
Men	11.219^*^	5			0.985	0.052	0.024			
Women	21.764^*^	5			0.985	0.064	0.020			
Configural	33.103^*^	10			0.985	0.060	0.020			
Metric	38.383^*^	14	3.952	4	0.984	0.052	0.032	−0.001	−0.008	0.012
Scalar	45.330^*^	18	5.459	4	0.982	0.049	0.038	−0.002	−0.003	0.005
**Models for age**	**χ** ^2^	**df**	**Δ*χ*^2^**	**Δgl**	**CFI**	**RMSEA**	**SRMR**	**ΔCFI**	**ΔRMSEA**	
Under 25	14.409^*^	5			0.991	0.047	0.017			
26–59 (adults)	8.652	5			0.992	0.042	0.020			
Configural	22.993^*^	10			0.991	0.045	0.018			
Metric	25.145^*^	14	0.628	4	0.992	0.035	0.021	0.001	−0.010	0.003
Scalar	28.236^*^	18	1.436	8	0.993	0.030	0.021	0.001	−0.005	0.000

*df, degrees of freedom; Δχ^2^, Chi Square increase; Δgl, increase in degrees of freedom; CFI, comparative fit index; RMSEA, root-mean-square error of approximation; SRMR, standardized root-mean-square residual; ΔCFI, CFI increase; ΔRMSEA, RMSEA increase; ΔSRMR, SRMR increase*.

* *p <0.001*.

Regarding validity, the SWLS presented statistically significant correlations (*p* < 0.001) that went in the expected direction regarding the other well-being variables (SPANE-P) (*r* = 0.603), SPANE-N (*r* = −0.376), Flourishing (*r* = 0.492), Optimism (*r* = 0.566) and Pessimism (*r* = −0.131). Finally, Table 4 shows the descriptive statistics and percentiles for each age group and gender.

**Table 4:** Descriptive statistics and percentiles for the satisfaction with life scale (SWLS) by age group and by gender.

		**Teens/emerging adults (*N* = 817)**	**Adults (*N* = 405)**	**Male (*N* = 435)**	**Female (*N* = 787)**
Mean		18.86	19.66	19.24	19.06
Median		19	20	20	20
Mode		20	25	20	20
Standard deviation		4.028	4.168	3.769	4.259
Skewness		−0.545	−0.843	−0.366	−0.722
SE of Skewness		0.086	0.121	0.117	0.087
Kurtosis		0.069	0.773	−0.235	0.339
SE of Kurtosis		0.171	0.242	0.234	0.174
Minimum		5	5	5	5
Maximum		25	25	25	25
Percentiles	5	12	12	13	11
	10	13	14	14	13
	15	15	15	15	15
	20	16	17	16	16
	25	16	17.5	17	16
	30	17	18	17	17
	35	17	18	18	18
	40	18	19	18	18
	45	19	19	19	19
	50	19	20	20	20
	55	20	20	20	20
	60	20	21	20	20
	65	21	22	21	21
	70	21	22	22	22
	75	22	23	22	22
	80	23	24	23	23
	85	23	24	24	24
	90	24	25	24.4	24
	95	25	25	25	25

*SE, standard error*.

## Discussion

It is suggested that “One reason for the increasing need for short scales could be a changing way to approach psychological research in general. With research questions becoming more and more complex, involving more and more constructs…” (78). Therefore, the effort to obtain valid instruments to assess well-being that are short and easy to answer is worth it to the point that a version of the SWLS with only three items has already been proposed (79). In the present work, the effort was aimed at evaluating the validity of the SWLS with fewer response options in order to facilitate its application in the Colombian context and offer data that facilitate researchers, clinicians, and, in general, professionals interested in the study of well-being to work on interpretation for evaluation purposes and in the design of interventions.

Despite the importance of how the person is asked to scale their response to a question or statement, there is little consensus in the literature regarding the number of points to include on a Likert response scale. Longer response scales have been suggested as preferable because they will increase variability in total scores and therefore would maximize precision and validity (80–83). However, what is important is that the variation in the scores allows for distinctions between individuals on the psychological characteristic that is evaluated. As early as the middle of the last century, Bendig (84) reported the same reliability for three, five, six, or nine answer options, but a decrease in reliability for 11 options. More recently, various studies (29, 85–88) have concluded that many response options can cause difficulties among participants in perceiving differences between alternatives written in a similar way (for example, agree vs. moderately agree), and induce biases in attributing lower numerical values to variables associated with social inequities and gender (89).

Our study shows that the validity of the SWLS is not affected by a reduction in the number of response options. In fact, this version shows excellent psychometric properties, including evidence of construct and concurrent validity with other measures related to well-being, which coincides with numerous studies carried out in various countries with different variants of the SWLS (18, 20–22, 90) and specifically in the Ibero-American context, with scales with a different number of response options, for example, in Spain (32, 33, 35–37, 91), in Chile (45, 92), in Peru (93), in Mexico (42, 94), in Puerto Rico (41) and in Argentina (95). In this sense, it will be necessary, in future studies, to determine the optimal number of response options, for which it may be useful to work using the Item Response Theory (IRT) so as to delve into the invariant properties of items and optimize the comparison of the results of the scale in different populations, as it is possible that other scales work better in different cultures (31, 83, 96). The use of IRT models in the study of response options can be done by estimating models that do not assume an order in the response categories (such as the nominal Bock model or the rating scale model), which allow estimating a location parameter for each of these response categories. This would allow us to check if the order of the response options is presented as it is assumed when dealing with items with a Likert-type response scale, or if said order is altered (97, 98). Likewise, these location parameters would allow us to check if each answer option has the maximum probability of being chosen for certain values in the trait. In the event that an answer option was less likely to be chosen than other adjacent options, this would indicate that said answer option has no relevance, since people would always prefer to choose one of the adjacent categories. This has been proven in various studies, in which the intermediate category is much less likely to be chosen than the adjacent categories (98, 99). And this can happen depending on the verbal anchor of the intermediate category.

Cross-cultural studies have confirmed the invariance of the unifactorial structure among nations, and initially enabled the detection of large differences that were attributed to sociocultural and socio-economic factors, such as national wealth and democratic governance (100). More recently, cross-cultural research revealed that these differences are also due to how different populations make judgments about satisfaction with life. For example, Emerson et al. (24) conducted a literature review on the cross-cultural invariance of the SWLS that encompassed works published in the last 30 years and included a sample of 27 articles with data from 66,380 respondents across 24 nations. This review corroborated the unifactorial structure of the scale, as well as the invariance by gender, but showed that there was no invariance between age groups and cultures. Similarly, other authors examined the invariance across 26 countries using three different methods, consistently finding configural and metric invariance, but not scalar invariance (101).

In our study, overall gender invariance was found, which was also confirmed for the seven-response option version in the Colombian population, and the total invariance among the youngest population (adolescents and emerging adults) and established adults (47). In this sense, our results are in line with those reported by other investigations with Spaniards (12, 91, 102), as well as with studies in other populations (103–105). However, it differs from other research that has not confirmed age invariance, such as a study carried out in Norwegian population (106). Contradictory results may be explained not only by specific cultural characteristics, but also by the characteristics of the samples, which is why this is a topic that should be studied in depth in the future.

As has been reported in studies with the SWLS around the world, the values of the items above the midpoint of the scale predominate (51). In addition, in our study, we identified differences in satisfaction with life attributable to age, consistent with the results reported by numerous studies (107). However, we did not find differences related to gender. According to Joshanloo and Jovanović (108), research on the relationship between satisfaction with life and gender has shown inconsistent results both in national studies and in large international studies and meta-analyses, since it can be influenced by various moderators such as sociocultural conditions, income, education, and marital status, among others (109). In Colombia, some studies have found that women have less satisfaction with life than men (110, 111) but it has been evaluated indirectly, based on data derived from econometric analyses. In any case, it is a topic that should continue to be studied, considering the limitations of the present study.

## Limitations

The scope of the results of our study is limited by the type of non-probability sampling used, as the sample was selected because of its accessibility, which restricts generalization, considering the cultural diversity of Colombia. In addition, the sample differs in the proportion in which age is distributed in the Colombian population. Considering the data from the last National Population and Housing Census of Colombia, our sample presents a higher proportion of young adults, as 82% of participants were under 30 years of age, and <1% of participants was above 65 years. However, nationally, those over 65 represent around 9% of the population, while young people between 18 and 30 years constitute ~16%. Similarly, in our sample, there are more people with a higher educational level than in the population, and it does not include any illiterate people, even though 5.9% of Colombians cannot read or write. Additionally, the online administration restricted participants by allowing only people with Internet access. In this sense, the psychometric properties of the scale should be studied in other populations, such as the rural population, and include representatives from the various ethnic groups that inhabit the national territory. Likewise, for future studies, temporal stability with a test-retest strategy is recommended.

## Conclusions

The five-choice SWLS maintains the excellent psychometric properties of the seven-choice version, with the advantage of being easier to answer. In addition, it presents invariance by gender and age groups, and provisional normative data are offered.

## Data Availability Statement

The raw data supporting the conclusions of this article will be made available by the authors, without undue reservation.

## Ethics Statement

The studies involving human participants were reviewed and approved by Comité de Bioética de Investigación de la Universidad Cooperativa de Colombia. The patients/participants provided their written informed consent to participate in this study.

## Author Contributions

MM-C, IC, and BE: conceptualization, methodology, and writing—review and editing. IC and BE: data curation. MM-C and BE: writing—original draft preparation. YP and MF-D: collect data in Santa Marta. AA and AC: collect data in Bucaramanga. JH: collect data in Antioquia. MM-C: project administration. All authors have read and agreed to the published version of the manuscript.

## Funding

The research data were part of a broader study, Project: An Analysis of the Psychometric Proper-ties of Diener's Well-Being Scales among the Colombian Population, funded by the Cooperative University of Colombia, in cooperation with the University of Valencia (Spain) and the Pontifical Bolivarian University of Bucaramanga, Grant Number INV2371.

## Conflict of Interest

The authors declare that the research was conducted in the absence of any commercial or financial relationships that could be construed as a potential conflict of interest.

## Publisher's Note

All claims expressed in this article are solely those of the authors and do not necessarily represent those of their affiliated organizations, or those of the publisher, the editors and the reviewers. Any product that may be evaluated in this article, or claim that may be made by its manufacturer, is not guaranteed or endorsed by the publisher.
